# Web alert: Microbial biofilm catalysis

**DOI:** 10.1111/1751-7915.14299

**Published:** 2023-06-20

**Authors:** Lawrence P. Wackett, McKnight

**Affiliations:** ^1^ Department of Biochemistry, Molecular Biology & Biophysics, BioTechnology Institute University of Minnesota St. Paul Minnesota USA

Biofilms and continuous chemical catalysis

https://www.sciencedirect.com/science/article/pii/S0167779912000741

This article discussed biofilms as a mechanism for production of chemicals, particularly fine chemicals. It has extensive coverage of methods for analysing biofilms.

Catalytic biofilms

https://www.ufz.de/index.php?en=42201

This is the page of an academic group studying catalytic biofilms.

Engineered biofilm catalysts

https://gtr.ukri.org/projects?ref=BB%2FI006834%2F1

This page describes a project to use engineered biofilms for the production of fine chemicals and pharmaceuticals.

*Pseudomonas* biofilm biotransformations

https://www.jmb.or.kr/journal/view.html?volume=23&number=2&spage=195

This article describes a *Pseudomonas* biofilm bioreactor that catalysed the conversion of ethylene glycol to glycolic acid over 2 months of continuous operation.

Polymer biofilms for biocatalysis

https://www.ncbi.nlm.nih.gov/pmc/articles/PMC9528183/

In some cases, the most catalytically useful bacteria are not adept at forming biofilms naturally. This study addressed that issue by demonstrating different methods of making polymer‐induced biofilms.

Anammox biofilm reactor

https://pubs.acs.org/doi/10.1021/acsestwater.0c00107

Anammox microbes have been shown to be very useful in industrial nitrogen removal from wastewaters. This paper describes the development and operation of a flow‐through Anammox biofilm reactor.

Trickle‐bed production of acetic acid

https://www.frontiersin.org/articles/10.3389/fenrg.2022.842284/full

An industrially useful biotechnological process involves the use of acetogenic bacteria to convert inexpensive gaseous chemicals into acetic acid. This paper describes a trickle‐bed reactor designed for this purpose.

Chemical imaging in biofilms

https://pubs.acs.org/doi/10.1021/acs.est.2c05027

This report describes sensitive mass spectrometric methods for imaging chemicals within biofilms.

Contaminant effects on biofilms

https://www.frontiersin.org/articles/10.3389/fmicb.2020.526545/full

This study examined the effects of nutrients and pharmaceuticals on biofilms.

Biofilms in the biorefinery

https://www.sciencedirect.com/science/article/pii/S0734975021000720

This review describes the use of the natural ability of microbes to form biofilms in wastewater treatment and large‐scale chemical conversions into products.

Productive biofilms

https://onlinelibrary.wiley.com/doi/full/10.1002/jctb.7208

This excellent review article describes the most industrially relevant microorganisms and reactor designs for productive industrial biofilm conversions.

